# Ammonia Increases the Stress of the Amazonian Giant *Arapaima gigas* in a Climate Change Scenario

**DOI:** 10.3390/ani13121977

**Published:** 2023-06-14

**Authors:** José Fernando Paz Ramírez, Renan Diego Amanajás, Adalberto Luis Val

**Affiliations:** 1 Programa de Pós-Graduação em Aquicultura, Universidade Nilton Lins, Avenida Professor Nilton Lins, 3259, Parques das Laranjeiras, Manaus CEP 69058-030, Brazil; josefernandopaz@gmail.com; 2 Laboratório de Ecofisiologia e Evolução Molecular, Instituto Nacional de Pesquisas da Amazônia, Avenida André Araújo, 2936, Petrópolis, Manaus CEP 69067-375, Brazil; dalval@inpa.gov.br; 3 Programa de Pós-Graduação em Biologia de Água Doce e Pesca Interior, Instituto Nacional de Pesquisas da Amazônia, Avenida André Araújo, 2936, Petrópolis, Manaus CEP 69067-375, Brazil

**Keywords:** Amazon, growth, climate, pirarucu, nitrogen residue

## Abstract

**Simple Summary:**

The world has been changing, and these changes have affected fish in the wild and in captivity. The Intergovernmental Panel on Climate Change (IPCC), which is an international body, predicts climate scenarios for the future that are based mainly on rising temperatures and carbon dioxide levels. Many species are sensitive to scenarios that are considered drastic, which suggests future losses in the rearing of aquatic organisms. In our study, we evaluate how these drastic climate scenarios and exposure to a sublethal concentration of ammonia, a product resulting from the consumption of dietary protein that can be toxic, affect the pirarucu *Arapaima gigas*, a carnivorous Amazonian fish species that can reach up to 12 kg in one year of farming, but needs to consume a large amount of protein in its feed to do so. Our results show that the pirarucu is sensitive to ammonia and climate change, and that this can impair the animal’s growth and some vital body functions, implying economic losses for those who produce it and higher prices for those who consume it.

**Abstract:**

Ammonia is toxic to fish, and when associated with global warming, it can cause losses in aquaculture. In this study, we investigated the physiological and zootechnical responses of *Arapaima gigas* to the current scenarios and to RCP8.5, a scenario predicted by the IPCC for the year 2100 which is associated with high concentrations of environmental ammonia (HEA). Forty-eight chipped juvenile *A. gigas* were distributed in two experimental rooms (current scenario and RCP8.5) in aquariums with and without the addition of ammonia (0.0 mM and 2.44 mM) for a period of 30 days. The HEA, the RCP8.5 scenario, and the association of these factors affects the zootechnical performance, the ionic regulation pattern, and the levels of ammonia, glucose, triglycerides, sodium, and potassium in pirarucu plasma. The branchial activity of H^+^-ATPase was reduced and AChE activity increased, indicating that the species uses available biological resources to prevent ammonia intoxication. Thus, measures such as monitoring water quality in regard to production, densities, and the feed supplied need to be more rigorous and frequent in daily management in order to avoid the accumulation of ammonia in water, which, in itself, proved harmful and more stressful to the animals subjected to a climate change scenario.

## 1. Introduction

Management during captive fish breeding usually causes stress which, when associated with environmental changes, puts productivity at risk. Despite the growth performance in aquaculture for human nutrition and food security, it is being affected by factors such as global warming, pollution, inappropriate land use, and hydrological changes [1]. High greenhouse gas emissions, such as increased carbon dioxide (CO_2_) levels that decrease environmental pH levels, result in rises in the Earth’s temperature and facilitate the occurrence of extreme weather events [2]. The inadequate management of breeding environments may favor the generation of other greenhouse gases, mainly due to the intensification of fish production, the use of high storage densities, and the use of high protein feed [3]. According to these authors, the metabolism of nitrogen compounds gains prominence in the face of the generation of products that can affect water quality, fish performance, and the dynamic balance of the environment, as is the case for nitrous oxide (N_2_O) and ammonia.

Ammonia (NH_3_ + NH_4_^+^) is the main product of protein metabolism [4]. In an aqueous solution, ammonia can be found in the form of gas (NH_3_, ammonia) and as a cation (NH_4_^+^, ammonium), which is commonly found in the body compartments of fish. The equilibrium equation is described as NH_3_ + H_3_O^+^ ⇔ NH_4_^+^ + H_2_O, the pK (cologarithm of an ionization constant) of the NH_3_/NH_4_^+^ reaction is about 9.0–9.5 [5], and toxicity increases at a high pH [6]. Fish excrete ammonia in the form of NH_3_ through the gill membranes in a metabolome complex that is regulated by rhesus-type glycoproteins (Rh), which involves the activity of proton pumps (H^+^-ATPases), Na^+^-ATPases, and exchangers such as Na^+^/H^+^ [4,7,8,9]. However, environments with high ammonia concentrations (HEA) hinder excretion gradients and cause accumulation in a fish’s body by taking in ammonia from the environment due to the decrease in the partial pressure gradient for NH_3_ and/or the electrochemical gradient for NH_4_^+^ [6]. 

Furthermore, the rise in temperature increases the metabolic rate of fish, enabling them to catabolize more proteins. This process generates more ammonia as a result of the nitrogen metabolism, which causes a decrease and a change in the pK of the ammonia equilibrium reaction, and thus, increases the NH_3_ fraction. This means there is more ammonia in the water, which can impair excretion [7,8,10]. Studies show that ammonia causes decreased growth, anemia, increased stress levels and susceptibility to disease, changes in morphology and branchial ionic regulation, muscle depolarization, blood dysfunctions, seizures, and death [11,12,13,14,15,16,17]. In the brain, ammonia can provoke edema, excitability, and swelling, but a system based on glutamine synthesis from glutamate and NH_4_^+^ prevents the intoxication of the brain and death of the fish [18]. 

The Intergovernmental Panel on Climate Change (IPCC), which is an international body, predicts climate scenarios for the future that are based mainly on rising temperatures and higher carbon dioxide levels. According to the 5th IPCC report, temperatures may increase by up to 4.8 °C and CO_2_ levels could reach over 1000 ppm [19]. Many species are sensitive to scenarios that are considered drastic, which suggests future losses in the rearing of aquatic organisms. The climate changes predicted by the IPCC (2014) [19] have already been shown to negatively affect the zootechnical performance and feed efficiency of Amazonian fish such as the tambaqui [20]. In more acidic environments, the efficient excretion of ammonia is expected; meanwhile, in warmer temperatures or at high environmental ammonia levels, the impairment of excretion can be expected [10,18]. 

Studies on the effects of ammonia on Amazonian fish are scarce, but are necessary in order to determine the impact of pollution and anthropization on aquatic environments, and to develop management practices for the good zootechnical performance of captive species. Although it has been hypothesized that Amazonian fish are tolerant of ammonia due to the hydrogeological characteristics of the basin, the work of Souza-Bastos et al. (2017) [16] indicates otherwise. In an acute exposure experiment with 11 species that were compared with species from other hydrographic regions, Amazonian species were observed to have greater sensitivity, with NH_4_Cl concentrations ranging from 2.5 to 40 mM. Other studies have shown that excretion can be seen as inversely proportional to temperature, with excretion increases occurring in relation to the mass of the individuals as observed in tambaqui (*Colossoma macropomum*) [21]. The pirarucu (*Arapaima gigas*) is an emblematic Osteoglossiformes of the Amazon basin that can reach up to 3 m in length and can weigh more than 200 kg [22]. During ontogeny, the species undergoes modifications that allow for the transition from aquatic to aerial respiration [23]. This process is linked to changes in the branchial morphology that prevent ion loss, such as increases in the blood–water barrier; in the modification of the bladder as an organ for taking oxygen; and in the ion regulation and the metabolism of nitrogenous residues by the kidneys [23,24,25,26]. According to Souza-Bastos et al. (2017) [16], the lethal concentration of total ammonia (NH_3_ + NH_4_^+^) for 50% of the subjects in an acute toxicity assay (96 h LC_50_—LC: lethal concentration) is, on average, 4.88 mM and, for 10% of subjects (96 h LC_10_), it is around 2.28 mM at pH 7. Since they are carnivorous, the feeding of pirarucus is based on feed with a protein content between 43% and 48% [27], and a better feed efficiency is achieved at high stocking densities when pirarucus are reared in small-volume tanks (120 m^3^) [28,29]. Four daily meals to apparent satiety seem to support a better mass gain given the high protein turnover of the species [30]. However, this has implications for the elevation of ammonia levels in the water in which they are reared, according to the different feeding strategies used [31]. Thus, if there is a lack of adequate management of water quality in the situations described, the excretion of the fish will cause an increase in environmental ammonia and may induce elevations of the metabolite in the plasma of the specimens [32], thus causing other types of metabolic damage and impairing growth.

Herein, it is hypothesized that environments with high environmental concentrations of ammonia may worsen these effects on pirarucu, given the sensitivity observed by Souza-Bastos et al. (2017) [16]. Thus, the objective of this study was to evaluate the physiological, ionoregulatory, and zootechnical responses of pirarucus reared in ammonia-rich environments that are a part of the conditions cited in the climate change scenarios predicted by the IPCC for the year 2100.

## 2. Materials and Methods

### 2.1. Experimental Fish and Acclimation

All procedures adopted in this study followed the recommendations of the Brazilian Council for Animal Experimentation (CONCEA), and the study was approved by the Ethics Committee on Animal Use at INPA under protocol # 026/2015.

Juvenile pirarucus were acquired from a local producer and transported in a 1000 L transport box to the Laboratory of Ecophysiology and Molecular Evolution at the National Institute for Amazonian Research (LEEM/INPA), where the experiment was carried out. To avoid the occurrence of stress and ion loss, 3 g L^−1^ of NaCl was added to the water. The water in the transport box was aerated the whole time. In the laboratory, the animals were acclimated in polyethylene boxes with a capacity of 500 L and fed with commercial feed (0.8–1.0 mm; 45% crude protein; 9% fat) for a period of 30 days. During the acclimation period, the specimens were kept in tanks with constant aeration (dissolved oxygen: 5.11 ± 0.91 mg L^−1^), a temperature of 26.3 ± 1.0 °C, electrical conductivity of 62.01 ± 35.78 µS cm^−1^, pH of 6.11 ± 0.92, total ammonia concentration of 0.011 ± 0.002 mM, and nitrite concentration of 0.02 ± 0.001 mg L^−1^.

### 2.2. Experimental Design

The experiment was conducted in two experimental rooms, which were 25 m^3^ each, at LEEM/INPA. These buildings simulate the conditions foreseen by the IPCC (2014) [19] for the Amazon climate in real time. Each room reproduced the current scenarios (control conditions) and the extreme (RCP8.5—representative concentration pathway) conditions according to the 5th IPCC climate report for the year 2100. The climate control room reflected, in real time, changes in temperature and CO_2_ level in a forest area without human influence, while the extreme climate room was based on the representative concentration of the IPCC (RCP8.5), and reproduced increases of 4.5 °C and 850 ppm of CO_2_ above the values of the current scenario. Every day for 30 days, CO_2_ and air temperature levels were monitored in both of the experimental rooms every 2 min. The photoperiod was 12 h light–12 h dark, and humidity was defined as a derived condition.

Juvenile pirarucus (*n* = 48), with a mean body mass of 35.87 ± 9 g and an initial length of 18 ± 1.30 cm, were fitted with chips in the dorsal region and randomly distributed in 240 L experimental aquariums (*n* = 12 fish/aquarium; density 1.73 kg/m^3^) for both scenarios to test the effects of the inclusion and absence of high environmental ammonia (HEA) concentrations on their physiological and zootechnical responses for a period of 30 days. For this, two treatments were established: a control without the addition of ammonia in the water (Amm0.0) and a treatment that represented 50% of the 96 h LC_50_ for pirarucus (Amm2.44) with 2.44 mM of (NH_4_)_2_SO_4_, as described by Souza-Bastos et al. (2017) [16]. The chipped fish was considered the experimental unit. In each aquarium, a protective screen was attached to prevent the fish from jumping out of the water, which is common in this species. In addition, to prevent the rise of ammonia levels in the water, a system that captured and filtrated solids was coupled to an ultraviolet (UV) filter and adapted in order to eliminate microorganisms in the aquariums for all treatments. The total filtration of the system occurred with a flow rate of 10 L min^−1^ throughout the entire experimental period. The animals were fed four times a day until apparent satiety throughout the experiment. The food remains were collected, dried, and weighed to calculate food consumption and carry out a conversion. Every day at 10 a.m. and 5 p.m., 40% of the water volume of the aquariums was removed and replaced with new water. After each change of water, the total ammonia was measured and readjusted to 2.44 mM.

At the end of the experimental period, the fish were anesthetized with buffered tricaine methanesulfonate (MS-222 0.5 mg L^−1^ + NaHCO_3_ 1.5 mg L^−1^; Sigma-Aldrich, St Louis, MO, USA), weighed with the aid of a set of scales with an accuracy of 0.1 g (Marte BL3200H, Minas Gerais, Brazil) and measured with a 0.1 cm ichthyometer for performance evaluation. Immediately after taking the growth measurements, blood samples were collected via caudal vessel puncture using syringes containing lithium heparin to determine blood and biochemical parameters. The collected blood was stored in 2 mL Eppendorf tubes in melting ice. Subsequently, the animals were euthanized by brain–spinal concussion and the first branchial arch was collected in order to evaluate the activity of the proton pumps (H^+^-ATPases), whereas the brain was analyzed to evaluate the activity of the acetylcholinesterase (AChE) enzymes.

### 2.3. Water Quality

Dissolved oxygen (mg L^−1^), conductivity (μS cm^−1^), pH, and temperature (°C) levels were monitored every day using a YSI probe (YSI 60 and 85, USA). CO^2^ levels were measured using a total carbon analyzer (TOC ANALYZER APOLLO 9000, Ohio, USA) and values were expressed in ppm. Total ammonia levels (NH_3_ + NH_4_ − TA-N) were determined using the colorimetric method proposed by Verdouw et al. (1978) [33] and expressed in mmol L^−1^. Non-ionized ammonia (NH_3_ − UIA-N) was estimated using the formula NH_3_ = [NH_3_ + NH_4_^+^]/[1 + 10^(pKa-pH)^], where pKa = 0.09018 + 2729.92/(273 + T), and was expressed in mmol L^−1^ [34]. Nitrite was measured using the method described by Boyd and Tucker (1992) [35] and sodium (Na^+^) and potassium (K^+^) levels were analyzed by means of flame photometry (ANALYSER 910, Piracicaba, Brazil), with values expressed in µmol L^−1^.

### 2.4. Zootechnical Performance

The evaluation of the zootechnical performance of the pirarucu was performed by measuring and calculating the body mass gain (WG), feed intake (FI), apparent feed conversion (FCR), growth in centimeters (C), relative condition factor (CF), and production cost (PC) with the following formulas:

Body mass gain (WG, g) = final mass (g) − initial mass (g)

Feed intake per fish (FI, g day^−^^1^ per fish) = total feed intake in the period (g)/experiment days

Apparent feed conversion rate (FCR) = feed intake (g)/body mass gain (g)

Growth in centimeters (C, cm) = final length (cm) − initial length (cm)

Condition factor (CF) = (mass (g)/total length (cm)^3^) × 100

Production costs (USD) = FCR × USD 1 kg of feed.

### 2.5. Hematological Parameters

From the whole blood samples, the hematocrit levels (Ht, %) were determined using the microhematocrit technique [36], the hemoglobin levels (Hb, g dL^−1^) were determined using the cyanomethemoglobin method described by Van Kampen and Zijlstra (1961) [37], and the methemoglobin levels (Met-Hb, %) were calculated according to Benesch et al. (1973) [38]. Blood aliquots were diluted (1:200—blood-to-solution ratio) in a solution of formalin citrate (3.8 g of Na_3_H_5_O_7_·2H_2_O, 2.0 mL of 40% formaldehyde, and 100 mL of distilled H_2_O q.s.p) for the erythrocyte count (RBCs, 10^6^ cells per mm^3^ of blood^−1^) using an optical microscope (professional B5, Motic^®^, Schertz, TX, USA) with a magnification of 40×. The corpuscular constants, mean corpuscular volume (MCV, fL), mean corpuscular hemoglobin (MCH, pg), and the mean corpuscular hemoglobin concentration (MCHC, %) were calculated based on the Ht, Hb, and RBC levels according to the recommendations of Brow (1976) [39].

### 2.6. Biochemical and Ionic Parameters of Plasma

At the end of the experiment, plasma samples were obtained after centrifugation of the whole blood in a centrifuge (Eppendorf, 5430R, Hamburg, Germany) at 1834 RCF for 7 min, which was then stored at −80 °C until the analyses were performed. Glucose and triglyceride levels were measured with colorimetric enzymatic methods using In Vitro^®^ commercial kits (In Vitro Diagnóstica, Belo Horizonte, Brazil), following the manufacturer’s recommendations, and were adapted for reading with a microplate reader (Spectra max Plus, model 384, Molecular Devices^®^, San Jose, CA, USA) at 500 nm. The plasma levels obtained were expressed in mg dL^−1^. Lactate concentrations were measured after acidification of the plasma with HCIO_4_ at 8%, centrifugation at 1207× *g* for 10 min, and neutralization with KOH 6M, which was followed by re-centrifugation for three minutes at the same rotational speed. Plasma was pipetted into microplates with glycine buffer, β-nicotinamide adenine dinucleotide hydrate (N6522, Sigma-Aldrich, St. Louis, MO, USA) and L-lactate dehydrogenase (L2500, Sigma-Aldrich, CA, USA). The microplate was incubated at 37 °C for about 10 min. The reading was performed on a microplate reader (SpectraMax M, Molecular Devices, San Jose, CA, USA) at a wavelength of 340 nm. Concentrations were expressed as mg dL^−1^.

After dilution of the samples in Milli-Q^®^ water at proportions of 1:1000 and 1:250 (*v*:*v*—plasma-to-water ratio), the levels of Na^+^ and K^+^ were measured using an atomic absorption spectrometer (AAnalyst 800, PerkinElmer, Hopkinton, MA, USA). Concentrations are expressed in mmol L^−1^.

### 2.7. H^+^-ATPase and AChE Activities

H^+^-ATPase enzyme activity was measured according to Kultz and Somero (1995) [40]. The assay was based on the inhibition of H^+^-ATPases by n-ethylmaleimide (NEM, 2 mmol L^−1^) in a freshly prepared, mixed solution containing 30 mmol L^−1^ of imidazole, 45 mmol L^−1^ of NaCl, 15 mmol L^−1^ of KCl, 3 mmol L^−1^ of MgCl_2_, 0.4 mmol L^−1^ of KCN, 1 mmol L^−1^ of Na_2_ATP, 0.2 mmol L^−1^ of NADH, 0.1 mmol L^−1^ of fructose-1,6-bisphosphate, 2 mmol L^−1^ of phosphoenolpyruvate (PEP), 3 IU of pyruvate kinase (PK), and 2 IU of lactate dehydrogenase (LDH). An aliquot of the mixture without the inhibitor was used to measure the total activity of the ATPases. Gill samples were homogenized (1:10 *w/v*) in SEI buffer (pH 7.5) containing 150 mmol L^−1^ of sucrose, 50 mmol L^−1^ of imidazole, 10 mmol L^−1^ of EDTA, and 2.5 mmol L^−1^ of deoxycholic acid. The homogenates were centrifuged at 2000× *g* for 7 min at 4 °C. The assay was performed by combining 200 µL of the reaction mix (with NEM and without the inhibitor) and 5 µL of the homogenates. Absorbances were measured at 340 nm (Spectra Max Plus, model 384, Molecular Devices^®^, San Jose, CA, USA) for 10 min. The H^+^-ATPase activity was calculated as the difference between the total activity and activity with NEM, and was expressed as µmol ADP ^−1^ mg^−1^ protein h^−1^.

The acetylcholinesterase (AChE) activity in the brain was measured using the method of Ellman et al. (1961) [41]. Initially, brain samples were homogenized in phosphate buffer (0.1 M of 20% glycerol, pH 7.5) and centrifuged for 20 min at 12,000× *g* at 4 °C. The supernatant was used to measure AChE activity. Acetylthiocholine iodide (ATC) was used as a substrate and 5,5′-dithiobis (2-nitrobenzoic) acid (DNTB) as a color reagent. The kinetic activity of AChE was measured at 415 nm using a spectrophotometer (Spectra Max Plus, 384, Molecular Devices^®^, San Jose, CA, USA), and expressed as nmol min^−1^ mg protein^−1^.

### 2.8. Statistical Analysis

The data of this study are presented as mean ± standard error of the mean (SEM, *n* = 12). Before developing the analyses, the data were tested for normality and homoscedasticity using the package Sigma Plot 11.0 (Systat Software Inc., Chicago, IL, USA). When the parametric statistical assumptions were met, the data were analyzed using two-way analysis of variance (two-way ANOVA) with ammonia and climate scenario as factors. Tukey’s post hoc test was used to verify the contrast between means when differences in the ANOVA results were detected. Differences were significant when *p* < 0.05.

## 3. Results

### 3.1. Water Quality Parameters

The temperatures and concentrations of CO_2_ observed in the air remained within those estimated by the IPCC for the RCP8.5 scenario (2014). An average increase of 3.02 ± 0.82 °C and 856 ± 3.74 ppm of CO_2_ was observed in relation to the values recorded in the current scenario. As expected, the variations observed in the air of the climatic rooms were reflected in the water of the aquariums, as shown in Table 1. The total ammonia (NH_4_^+^ + NH_3_) increased in both scenarios after adding (NH_4_)_2_SO_4_ to the water (see Table 1).

The average values of the parameters of water quality during the experimental period are shown in Table 1. The observed oxygen levels were lower in the aquariums that had ammonia in both scenarios (*p* < 0.05). The mean values of electrical conductivity were higher in the two scenarios with the presence of ammonia in the water when compared to aquariums without ammonia (*p* < 0.05). There was an approximately 1.9- and 2.1-fold increase in non-ionized ammonia in the water of the scenarios without and with the presence of ammonia, respectively. Between the analyzed scenarios, the values recorded for pH, nitrite, sodium, and potassium in the water did not vary throughout the experimental period (*p* > 0.05).

### 3.2. Growth Performance

**Figure 1:**
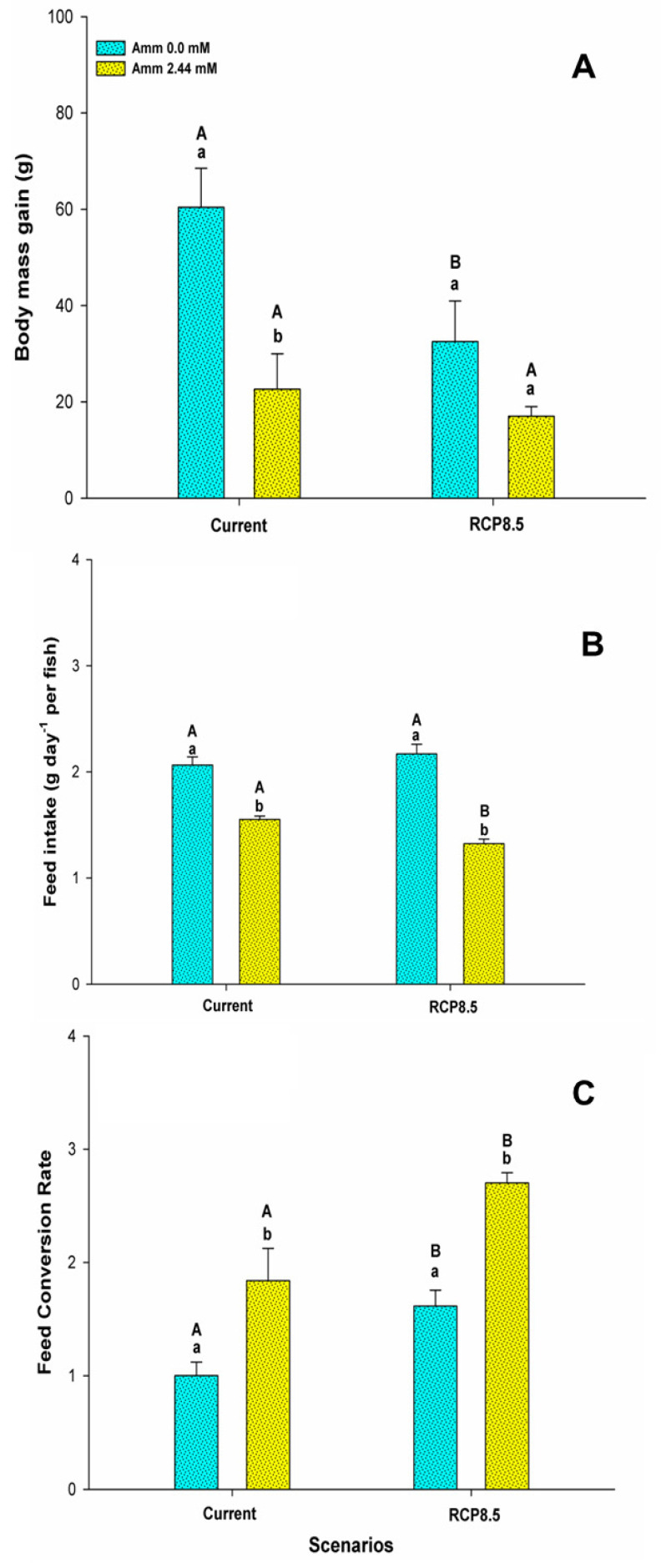
(**A**) Body mass gain (g), (**B**) food consumption (g day^−1^ per fish) and (**C**) feed conversion rate of the juvenile pirarucus (*n* = 12) in the current scenario and in the climate scenario predicted by the IPCC for the year 2100 with and without the presence of ammonia in the water. Lowercase letters indicate differences within the same scenario for animals reared with the presence or absence of ammonia in water, while uppercase letters indicate differences for animals reared under the same ammonia conditions in the water between the climate scenarios (*p* < 0.05).

**Figure 2:**
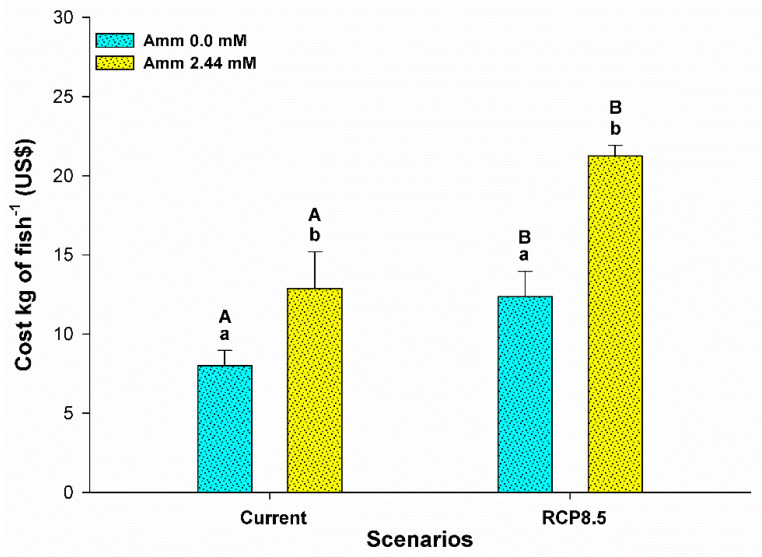
Average production cost per kg fish^−1^ (USD) for juvenile pirarucus (*n* = 12) in the current scenario and in the climate scenario predicted by the IPCC for the year 2100 with and without the presence of ammonia in the water. Lowercase letters indicate differences within the same scenario for animals reared with the presence or absence of ammonia in water, while uppercase letters indicate differences for animals reared under the same ammonia conditions in the water between the climate scenarios (*p* < 0.05).

No mortality was observed during the experimental period. The mass gain (WG) of the fish was influenced by ammonia (F = 14,557; *p* = 0.001) and by the climatic scenario (F = 5,752; *p* = 0.026), and there was no interaction effect between the factors (F = 2,547; *p* = 0.126) (Figure 1A). The presence of ammonia in the water resulted in a lower WG for the animals in the current scenario compared to the that for the animals in the scenario without the presence of ammonia in the water (*p* < 0.05). In the RCP8.5 scenario, the WG did not differ between the groups due to the presence of ammonia (*p* > 0.05), but the WG was lower in the RCP8.5 scenario group without the presence of ammonia in the water compared to the current scenario group without the presence of ammonia in the water (*p* < 0.05). Food consumption (FI) was influenced by the interaction between ammonia and the climate scenario (F = 6,456; *p* = 0.012). The presence of ammonia in the water resulted in lower food consumption in both climatic scenarios and for the animals of the RCP8.5 scenario in relation to those of the current scenario (*p* < 0.05) (Figure 1B). The feed conversion rate (FCR) was influenced only by ammonia (F = 38,693; *p* <0.001) and the climate scenario (F = 22,712; *p* < 0.001). Animals under the influence of ammonia in the water showed an increase in the FCR in both climatic scenarios (*p* < 0.05) (Figure 1C). The FCR was elevated in the RCP8.5 scenario compared to the current one for fish reared with or without the presence of ammonia in the water (*p* < 0.05). The growth in the length (cm) of the animals was influenced only by ammonia (F = 7,668; *p* = 0.012), while the condition factor (CF) was influenced by ammonia (F = 4,731; *p* = 0.042) and the climate scenario (F = 4,440; *p* = 0.049) (Table 2). For both parameters in the current scenario, the presence of ammonia in the water resulted in a lower performance (*p* < 0.05). There was also a decrease in these parameters for the animals in the RCP8.5 scenario in relation to the current one (*p* < 0.05). The production cost per kilogram of the species was influenced by ammonia (F = 28,464; *p* < 0.001) and the climatic scenario (F = 24,254; *p* < 0.001) (Figure 2). The presence of ammonia in the water in the current scenario increased the production costs of the animals (*p* < 0.05). In the RCP8.5 scenario, a higher production cost was observed in relation to the current scenario and was independent of ammonia levels in the water (*p* < 0.05).

### 3.3. Hematological Parameters

Throughout the experimental period, no variations were observed in the Ht, Hb, RBC, MCV, MCH, MCHC, and Met-HB values (Table 3) of the juvenile pirarucus reared under the current scenario and RCP8.5 scenario with or without ammonia in the water (*p* > 0.05).

### 3.4. Plasma Metabolites

**Figure 3:**
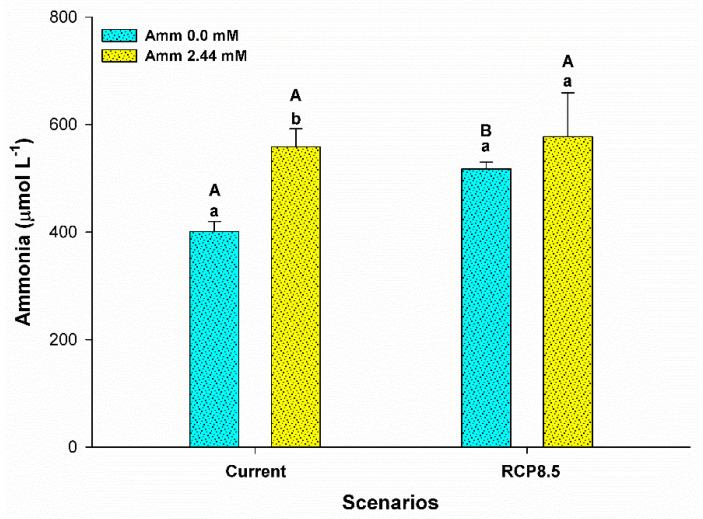
Plasma ammonia (µmol L^−1^) of the juvenile pirarucus (*n* = 12) in the current scenario and in the climate scenario predicted by the IPCC for the year 2100 with and without the presence of ammonia in the water. Lowercase letters indicate differences within the same scenario for animals reared with the presence or absence of ammonia in water, while uppercase letters indicate differences for animals reared under the same ammonia conditions in the water between the climate scenarios (*p* < 0.05).

**Figure 4:**
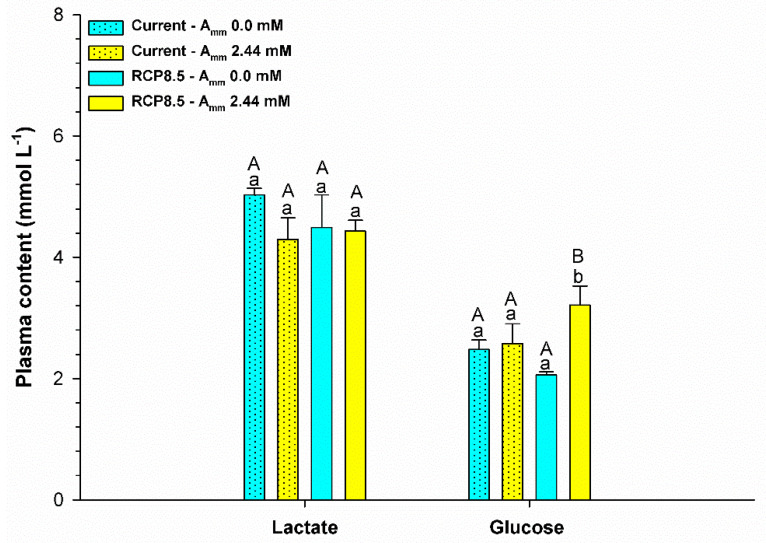
Plasma lactate and glucose (mg dL^−1^) of the juvenile pirarucus (*n* = 12) in the current scenario and in the climate scenario predicted by the IPCC for the year 2100 with and without the presence of ammonia in the water. Lowercase letters indicate differences within the same scenario for animals reared with the presence or absence of ammonia in water, while uppercase letters indicate differences for animals reared under the same ammonia conditions in the water between the climate scenarios (*p* < 0.05).

**Figure 5:**
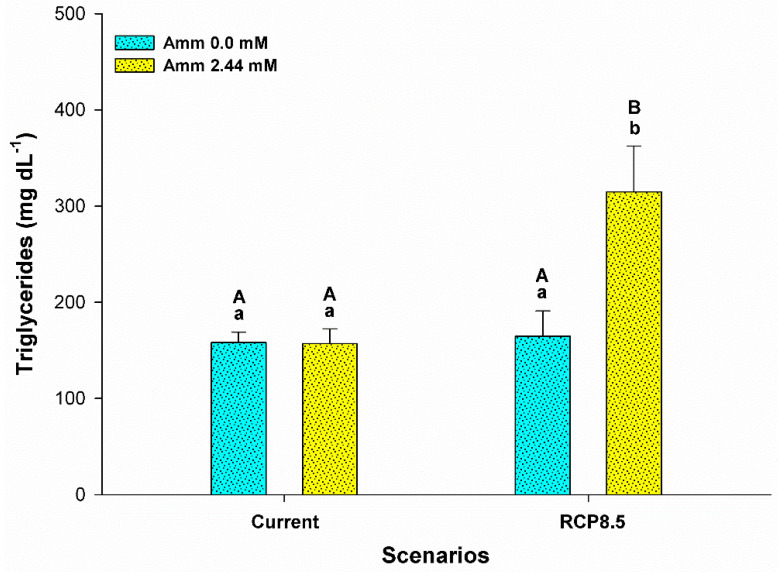
Plasma triglycerides (mg dL^−1^) of the juvenile pirarucus (*n* = 12) in the current scenario and in the climate scenario predicted by the IPCC for the year 2100 with and without the presence of ammonia in the water. Lowercase letters indicate differences within the same scenario for animals reared with the presence or absence of ammonia in water, while uppercase letters indicate differences for animals reared under the same ammonia conditions in the water between the climate scenarios (*p* < 0.05).

**Figure 6:**
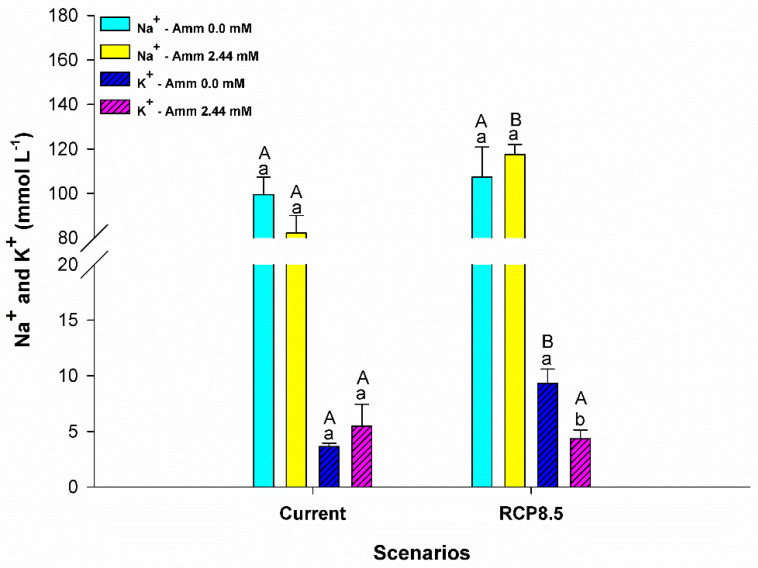
Sodium (Na^+^) and potassium (K^+^) in plasma (mmol L^−1^) of the juvenile pirarucus (*n* = 12) in the current scenario and in the climate scenario predicted by the IPCC for the year 2100 with and without the presence of ammonia in the water. Lowercase letters indicate differences within the same scenario for animals reared with the presence or absence of ammonia in water, while uppercase letters indicate differences for animals reared under the same ammonia conditions in the water between the climate scenarios (*p* < 0.05).

Ammonia levels in the plasma were influenced only by the level of ammonia in the water (F = 6,515; *p* = 0.024). In the current scenario, a higher concentration of plasma ammonia was observed for animals that had ammonia added to the water (*p* < 0.05), as shown in Figure 3. In addition, the RCP8.5 climatic scenario group showed that the animals reared without the addition of ammonia in the water demonstrated an increase in the levels of this metabolite (*p* < 0.05). Glucose levels (F = 7,626; *p* = 0.012) and triglycerides (F = 6,886; *p* = 0.016) were influenced by the interaction between ammonia and the climatic scenario. An increase in the levels of these metabolites was observed for animals that had ammonia added to the water in the RCP8.5 scenario and in the current scenario (*p* < 0.05) (Figure 4 and Figure 5). Plasma lactate levels did not differ between treatments (*p* > 0.05) (see Figure 4). Plasma sodium was affected by the climatic scenario (F = 5,135; *p* = 0.036). An increase in sodium levels was observed for animals reared in an environment with ammonia in the RCP8.5 scenario when compared to the current one (*p* < 0.05). Plasma potassium was affected by the interaction between the analyzed factors (F = 8,033; *p* = 0.011). Potassium levels showed a behavior that was contrary to sodium, with high levels for animals reared without the addition of ammonia in the water in the RCP8.5 scenario in relation to the current one (*p* < 0.05) (Figure 6).

### 3.5. Enzymes

**Figure 7:**
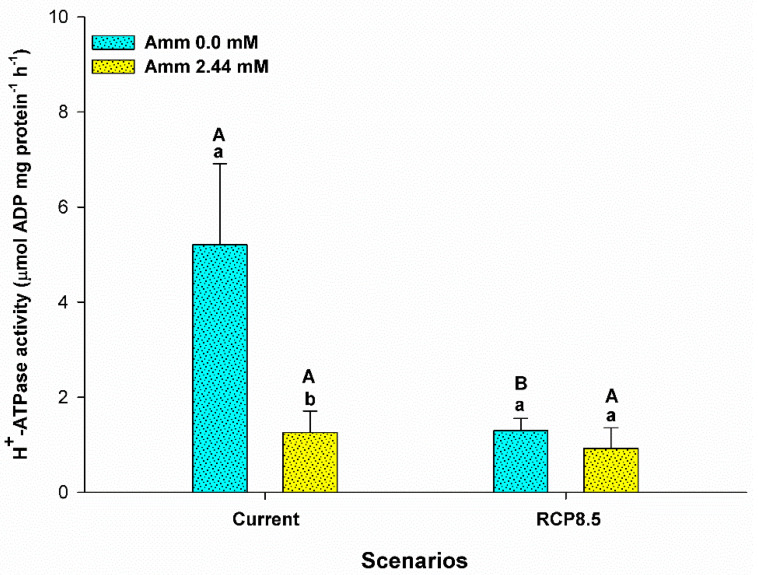
H^+^-ATPase activity in gills (µmol ADP mg protein^−1^ h^−1^) of the juvenile pirarucus (*n* = 12) in the current scenario and in the climate scenario predicted by the IPCC for the year 2100 with and without the presence of ammonia in the water. Lowercase letters indicate differences within the same scenario for animals reared with the presence or absence of ammonia in water, while uppercase letters indicate differences for animals reared under the same ammonia conditions in the water between the climate scenarios (*p* < 0.05).

The enzyme H^+^-ATPase was influenced by the interaction between ammonia and the climatic scenario (F = 10,582; *p* = 0.001) (Figure 7). H^+^-ATPase enzymatic activity was lower in animals reared in an environment with ammonia in the current scenario (*p* < 0.05). In addition, lower enzyme activity was observed in the RCP8.5 scenario compared to the current one for animals reared without the addition of ammonia in the water (*p* < 0.05).

**Figure 8:**
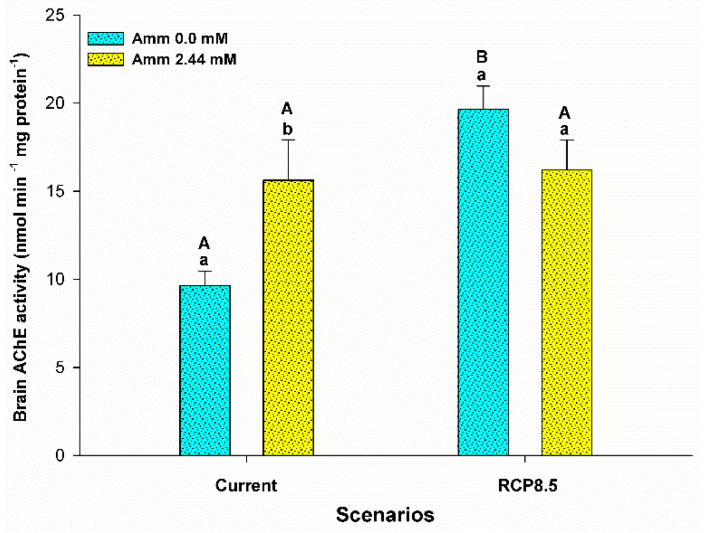
Brain acetylcholinesterase (AChE) activity (nmol min^−1^ mg protein^−1^) of the juvenile pirarucus (*n* = 12) in the current scenario and in the climate scenario predicted by the IPCC for the year 2100 with and without the presence of ammonia in the water. Lowercase letters indicate differences within the same scenario for animals reared with the presence or absence of ammonia in water, while uppercase letters indicate differences for animals reared under the same ammonia conditions in the water between the climate scenarios (*p* < 0.05).

The enzyme acetylcholinesterase (AChE) also was influenced by the interaction between ammonia and the climatic scenario (F = 8,349; *p* = 0.009) (Figure 8). In the current scenario, AChE activity was higher for animals reared with the addition of ammonia in the water (*p* < 0.05). On the other hand, in the RCP8.5 scenario, an increase was observed in relation to the current scenario for animals reared without the addition of ammonia in the water (*p* < 0.05).

## 4. Discussion

### 4.1. Water Quality

The variations in temperature levels and the air and water CO_2_ levels observed in this study were within the values estimated by the IPCC for the RCP8.5 scenario and are in agreement with the findings from other studies that have already been carried out [19,42,43]. The water quality assessment indicated an increase in electrical conductivity and a decrease in oxygen levels in the tanks in which ammonia was added in both scenarios. This increase in the levels of ammonia is associated with the dissociation of ammonium sulfate ([NH4]_2_SO_2_), which is caused by the insertion of the experimental concentration of ammonia to the water in the aquariums. A similar result was observed by Cavero et al. (2004) [32], who evaluated the effect of the confinement time of pirarucus in environments containing ammonia, in which the increase in electrical conductivity was proportional to the addition of ammonium chloride (NH_4_Cl) in the water of the experimental aquariums. Environments that contain ammonia cause an increase in oxygen consumption by fish and an increase in the rate of excretion of CO_2_ in an attempt to eliminate it from the body, which results in a decrease in O_2_ levels present in the water [44], as noted in this study. Under higher temperatures, these effects tend to increase, inducing animals to perform a greater excretion of ammonia as well [45] (see Table 1).

### 4.2. Growth

The results of this study show that the exposure of the pirarucu to ammonia and ammonia’s association with the extreme scenario of climate change affect the growth of the species. The excessive presence of ammonia in the water reduces mass gain and feed intake, and worsens feed conversion. These data reinforce the idea that significant concentrations of ammonia in the water lead to behavioral and physiological changes that negatively impact growth. We believe that the species reduces its feed consumption under HEA concentrations as a strategy to avoid ammonia accumulation in water, harming it even more. In this way, the growth and feed conversion rate were affected. This is an effect that has already been reported for species such as Atlantic cod (*Gadus morhua*) [46], goldfish (*Carassius auratus* L.) [8], marine medaka (*Oryzias melastigma*) [12], and spotted wolffish (*Anarhichas minor*) [47]. However, we expected that the exposure of pirarucus to UIA-N in the RCP8.5 scenario would result in a worsening of the mass gain, growth, and condition factor by the additional stressor effect of a rise in temperature and CO_2_ levels in this scenario (see Figure 1 and Table 2). The effects of the presence of ammonia were more evident on these variables than on the scenario. In relation to this, Cuenco et al. (1985) [48] emphasized that in environments with temperature manipulation, the presence of high concentrations of ammonia can decrease the sensitivity of animals to thermal variations in associated exposures. In fact, this can be observed for the pirarucu when comparing the RCP8.5 scenario for animals without the addition of UIA-N. Against the current scenario, we can observe a decrease in the variables, which characterizes the preponderant effect of the scenario with higher temperatures (see Figure 1 and Table 2). Thus, the species seems to be precocious in responding to the effect of the RCP8.5 scenario after 30 days of rearing, since a study conducted with tambaqui (*Colossoma macropomum*) under similar conditions highlighted changes in the performance of the species only after 90 days of rearing [20].

### 4.3. Production Costs

The price of fish is a measure that is capable of providing indicators about the costs of fisheries and farm production [49]. Given the ongoing changes in the climate, concern about the volatility and variation of these costs is directly linked to an increase in vulnerability and food security in the future. In this study, the rearing of pirarucus in the current scenario containing ammonia, as well as when subjected to the RCP8.5 scenario, indicated an increase in the production costs of the species that ranged from USD 2 to USD 3.4 per kg (Figure 2). According to Ferreira et al. (2020) [50], it is necessary to prioritize the production chain of this species with the aim of reducing costs, adding value, and identifying markets; however, these are topics with little coverage in the literature and that have numerous implications for the market price and rearing. According to Valladão et al. (2016) [51], values can reach up to USD 25 per kg on the international market. Notably, the data from this study suggest that chronic exposure to ammonia or a climate change scenario would result in increased costs per kg of fish compared to environmentally controlled scenarios. This would represent an increase in prices for the consumer and would require better evaluation of the environmental performance of fish during extended production cycles in which there is a low conversion of food and greater excretion into the water, since the species requires a diet containing high protein values during its rearing.

### 4.4. Hematological Parameters

The exposure of fish to ammonia has been associated with hematological alterations and increased energy expenditure due to the stress in relationships that are expressed as time-dependent or dose-dependent [14,15]. In this study, the pirarucus did not present variations in their hematological parameters under any of the evaluated conditions, which shows that the challenges caused by ammonia and the RCP8.5 scenario may not have induced or prolonged hematopoietic responses during the experimental period (Table 3). Our data differ from what has been reported in studies involving hypercapnia, the climate scenarios predicted by the IPCC, or for fish exposed to HEA in acute or chronic conditions [20,52,53,54]. In these studies, species-specific variations were observed in the Ht, Hb, RBC, MCV, and MCH levels. The effects of exposure to HEA in experiments conducted under elevated partial CO_2_ pressure have shown that there is a buffering effect of ammonia on the blood pH from which acidosis compensation is time-dependent, as was described for *Dicentrarchus labrax* [55], and that individuals decrease their gill ventilation rates by increasing air breathing, which may explain the non-observance of variations in hematology.

### 4.5. Plasma Ammonia, Plasma Ions, and H^+^-ATPase

Ammonia is toxic to most fish. High environmental concentrations of ammonia (HEA) contribute to a decrease in excretion gradients, thus favoring accumulation in the different body compartments of fish and interference in processes such as ion regulation and acid–base balance [18,56,57,58]. In this study, the exposure of pirarucus to scenarios containing ammonia resulted in an increase in plasma concentrations. Our data corroborate the findings of Zhang et al. (2019) [17] regarding the blunt snout bream species (*Megalobrama amblycephala*), in which an increase in ammonia levels was also observed after 28 days of exposure; additionally, our findings are in agreement with those of Nawata and Wood (2008) [59] for rainbow trout (*Oncorrhynchus mykiss*) exposed to environments with 1.5 mmol of NH_4_HCO_3_ and an increment of 1% of CO_2_ in the air. The increase in temperature and CO_2_ in the RCP8.5 scenario without ammonia in the water contributed to the increase in plasma levels of this metabolite in the pirarucu. The low conversion of food, given the maintenance of the feeding rate, may be a result of the increase in catabolism, which explains the worsening of performance and the increase in ammonia in these animals (see Figure 1B,C and Figure 3). Note that in the air-breathing mudskipper (*Periophthalmodon schlosseri*), the reduction in protein catabolism was a strategy used to prevent the accumulation of and poisoning by ammonia [60]. The increase in plasma ammonia in pirarucus, under the conditions described above, possibly correlate with the observed decrease in vH^+^-ATPase enzyme activity (Figure 7), since the enzyme is involved in the metabolome proposed for the excretion of ammonia (see Wright and Wood 2009 [4]), which may be favoring the buffering of pH by ammonia, its elevation, and, consequently, its plasma accumulation [55]. In this sense, our findings contrast with those of Almeida-Silva et al. (2020) [61] for the species *Pyrrhulina brevis*, which showed increased activity of this enzyme in the RCP8.5 scenario (high temperature + high CO_2_) compared to the current scenario, suggesting that in these animals ammonia may be eliminated more efficiently due to coupling caused by the capture of H^+^ that results from excretion via vH^+^-ATPase and the hydrolysis of CO_2_ by the enzyme carbonic anhydrase [62]. We also observed an increase in plasma Na^+^ and decrease in K^+^ in animals exposed to the RCP8.5 + ammonia scenario in water, suggesting an osmoregulatory disturbance in an attempt to eliminate ammonia (Figure 6), as was previously observed in Indian major carp (*Catla catla*) after 7 to 35 days of exposure to (NH_4_)_2_SOw_4_ [63]. Our findings suggest the possibility that the pirarucu performs the active excretion of ammonia via the Na^+^/NH_4_^+^ exchanger in the basolateral membrane via Na^+^-(NH_4_^+^)K^+^ATPase and still experiences a transcellular loss of K^+^, which would encourage the resumption of Na^+^ via the exchanger Na^+^/H^+^ in the metabolome facilitated by Rhesus proteins (see Wright and Wood 2009; [4,14,64]). Thus, our data highlight paths that reinforce the hypothesis proposed by Brauner et al. (2004) [23] for the species, the findings of Wood et al. (2014) [65] for cardinal tetra (*Paracheirodon axelrodi*) in HEA, and the basolateral detection of branchial Na^+^K^+^ATPase by Frommel et al. (2021) [66]. In this sense, not only the gills but the kidneys may also contribute to excretion, since the proportion eliminated via urine is higher than that eliminated via the gills, and the Na^+^/NH_4_^+^ exchangers have already been observed in the kidneys of the species [23,26].

### 4.6. Biochemical Parameters in Plasma

The increases in the levels of glucose (Figure 4) and triglycerides (Figure 5) indicate that animals exposed to the RCP8.5 + ammonia scenario suffer more stress, which suggests that, in an attempt to deal with ammonia and avoid intoxication, these animals decrease their feeding rates and start using their available energy reserves, as the protein turnover in the species is high and this would require greater energy [25]. In addition, data reported for goldfish indicate that animals fed over 28 days, a time similar to that of the present study, accumulate a greater amount of ammonia in the muscle, thus suggesting an increase in its toxicity to animals exposed to HEA [8]. However, in our study, anaerobic metabolism did not seem to predominate since the lactate levels remained invariable throughout the experimental period (Figure 4), which may reflect the adequate availability of oxygen for the animals.

### 4.7. AChE Activity

Excess ammonia in the brain caused by HEA can induce a disruption in nerve functions that results in increased excitability, a higher rate of ventilation, convulsions, coma, and death [56,67]. In order to deal with toxicity, fish can convert ammonia to less-toxic products via detoxifying enzymes [12]. On the other hand, the normal functioning of the brain is also dependent on enzymes that act on neuronal and neuromuscular transmission. AChE is an enzyme that hydrolyzes the acetylcholine molecule into choline and acetic acid in the synaptic cleft, and ensures adequate transmission of nerve impulses [68]. An impairment in its functioning can affect activities such as locomotion, feeding, balance, and swimming. Studies involving pollution and the use of pesticides are among the most evaluated topics regarding its analysis [69]. In this study, AChE activity in the brain (Figure 8) showed activity similar to that observed for ammonia concentrations (see Figure 3), which was higher in the groups that were exposed to ammonia, as well as in the group of animals that were under the changed scenario without ammonia in the water (Figure 8). These findings indicate that the exposure of pirarucus to ammonia and the RCP8.5 scenario exerts a stimulating effect on the nervous system in an attempt to prevent the accumulation of acetylcholine in the synaptic cleft, impacting vital functions. In the species mrigal (*Cirrhinus mrigala*), increasing exposure to different ammonia concentrations induced a decrease in AChE brain activity and caused behavioral changes in a 96 h acute experiment [70]. However, further studies are needed to investigate the effect on the regulation of this enzyme in fish exposed to ammonia.

## 5. Conclusions

The data of the present study show that, contrary to its indicated rusticity, the pirarucu is a species that is relatively sensitive to ammonia. Rearing the species under chronic ammonia conditions in water and in an extreme climate scenario could further affect performance due to rising production costs.

The impact on osmoregulation needs to be better evaluated, although the data suggest the possibility of impairments and the involvement of a complex network of interactions to ensure ammonia excretion. It is necessary to strongly control and manage the quality of the water in which it is reared, since the species requires high levels of protein in the diet and develops better in high storage densities. Thus, productivity can be high and will ensure better growth and zootechnical performance of the pirarucu.

## Figures and Tables

**Table 1:** Water quality for the current scenario and RCP8.5 scenario in aquariums containing pirarucu (*A. gigas*)^.1^ with and without ammonia.

Parameter	Current	Current + A_mm_ 2.44	RCP8.5	RCP8.5 + A_mm_ 2.44
Temperature (°C)	27.5 ± 0.2 ^aA^	27.7 ± 0.2 ^aA^	29.9 ± 0.1 ^aB^	29.6 ± 0.1 ^aB^
CO_2_ (ppm)	6.2 ± 0.05 ^aA^	4.8 ± 0.4 ^aA^	9.3 ± 0.5 ^aB^	11 ± 0.9 ^aB^
pH	5.81 ± 0.11	5.46 ± 0.16	5.86 ± 0.11	5.76 ± 0.14
OD (mg·L^−1^)	6.13 ± 0.12 ^aA^	5.69 ± 0.14 ^bA^	6.17 ± 0.1 ^aA^	5.55 ± 0.1 ^bA^
EC (µS·cm^−1^)	57.63 ± 2.02 ^aA^	376.81 ± 10.37 ^bA^	55.52 ± 2.98 ^aA^	392.03 ± 10.03 ^bA^
NH_4_^+^ + NH_3_ (mmol·L^−1^)	0.15 ± 0.01 ^aA^	2.6 ± 0.1 ^bA^	0.18 ± 0.01 ^aA^	2.6 ± 0.1 ^bA^
NH_3_ (mmol·L^−1^)	1.29 × 10^−4^ ± 0.29 × 10^−4 aA^	16.70 × 10^−4^ ± 3.7 × 10^−4 bA^	2.26 × 10^−4^ ± 0.45 × 10^−4 aA^	34.30 × 10^−4^ ± 7.70 × 10^−4 bB^
NO_2_ (mmol·L^−1^)	0.023 ± 0.005	0.024 ± 0.006	0.023 ± 0.005	0.024 ± 0.005
Na^+^ (µmol·L^−1^)	53.45 ± 3.07	52.51± 3.62	57.21 ± 4.04	59.1 ± 3.47
K^+^ (µmol·L^−1^)	14.54 ± 0.98	14.50 ± 1.12	15.81 ± 1.13	12.62 ± 0.92

^1^ The values are shown as mean ± SEM (*n* = 12). Lowercase letters indicate differences between tanks with and without ammonia in the same scenario, while uppercase letters indicate differences between scenarios with the same ammonia concentration (*p* < 0.05).

**Table 2:** Effects of ammonia on growth (cm) and condition factor in juvenile pirarucus (*n* = 12) reared under the current scenario and RCP8.5 scenario with and without the presence of ammonia in the water ^1^.

Scenario				
Parameter	Current	Current + A_mm_ 2.44	RCP8.5	RCP8.5 + A_mm_ 2.44
Growth (cm)	8.20 ± 0.67 ^aA^	4.53 ± 0.83 ^bA^	5.31 ± 0.94 ^aB^	4.61 ± 0.67 ^aA^
Condition factor (CF)	1.75 ± 0.03 ^aA^	1.60 ± 0.04 ^bA^	1.60 ± 0.05 ^aB^	1.59 ± 0.02 ^aA^

^1^ The values are shown as mean ± SEM (*n* = 12). Lowercase letters indicate differences between tanks with and without ammonia in the same scenario, while uppercase letters indicate differences between scenarios with the same ammonia concentration (*p* < 0.05).

**Table 3:** Hematological profile of juvenile pirarucus (*n* = 12) reared per the current scenario and RCP8.5 scenario with and without the presence of ammonia in the water ^1^.

Scenario				
Parameter	Current	Current + A_mm_ 2.44	RCP8.5	RCP8.5 + A_mm_ 2.44
Ht (%)	35.8 ± 1.1	36.4 ± 1.2	32.8 ± 1.7	35.6 ± 1.1
Hb (g·dL^−1^)	10.45 ± 0.46	11.26 ± 0.46	10.29 ± 0.46	11.16 ± 0.46
RBC (10^6^ cells·mm^3^)	2.05 ± 0.10	2.26 ± 0.10	2.04 ± 0.10	2.13± 0.09
MCV (fL)	177.89 ± 7.92	164.62 ± 7.92	161.56 ± 7.92	168.45 ± 7.92
MCH (pg)	51.16 ± 2.91	50.61 ± 2.91	51.18 ± 2.91	52.46 ± 2.91
MCHC (%)	28.9 ± 1.3	30.8 ± 1.3	31.6 ± 1.3	31.3 ± 1.3
Met-Hb (%)	8.4 × 10^−4^ ± 0.4 × 10^−4^	7.5 × 10^−4^ ± 0.4 × 10^−4^	7.7 × 10^−4^ ± 0.4 × 10^−4^	8.1 × 10^−4^ ± 0.4 × 10^−4^

^1^ The values are shown as mean ± SEM (*n* = 12).

## Data Availability

The data presented in this study are available upon request from the corresponding author.
